# The Growth of Municipal Sanatoria: Their Administration, Work, and Aims

**Published:** 1913-09-20

**Authors:** Frederick J. C. Blackmore

**Affiliations:** Senior Assistant Medical Officer to the City Sanatorium and Tuberculosis Centre, Birmingham.


### THE GROWTH OF MUNICIPAL SANATORIA.

### Their Administration, Work, and Aims.

By FREDERICK J. C. BLACKMORE, M.R.C.S., L.R.C.P., Senior Assistant Medical Officer to the City Sanatorium and Tuberculosis Centre, Birmingham.

The provision of sanatoria, rendered necessary by the adoption of the Report of the Departmental Committee, is now being considered by many municipal authorities. There is very little to guide them in the experience of other municipalities. This question, for instance, has not arisen in Germany. Owing to the comparatively recent growth of a Public Health Service in that country, the workmen's societies and sickness insurance societies themselves found it necessary to provide some means of treating such of their members as were suffering from tuberculosis. The erection of sanatoria in Germany has been largely due to these societies; and they have found that, in spite of the heavy initial expense, there has been an appreciable saving of their resources.

The City of Birmingham was the first municipality entirely to own and manage a sanatorium as a part of its organisation in the prevention and cure of tuberculosis. Dr. Bodington, who initiated the theory and practice of the open-air treatment, was a Birmingham man, and the first English sanatorium was started at Sutton Coldfield, now a suburb of that City. It is, therefore, peculiarly appropriate that Birmingham should have led the way in providing municipal sanatoria. As the service is now well organised, it will be advantageous to consider very shortly what has been done in the way of providing institutional treatment. It was soon recognised that it was impossible to expect charitable and semi-charitable institutions to provide for the needs of such a large population. The Corporation therefore purchased, in 1907, the estate of Salterley Grange, near Cheltenham. This comprised a large mansion and 380 acres of land. Part of the land was let off, and only thirty acres retained surrounding the house. The latter was utilised as the administrative block, and four pavilions for patients were erected. These are on the cubicle system; two pavilions contain sixteen rooms each, and two contain four rooms each. On October 15, 1908, the building was formally opened for the reception of patients. The demand for beds was so great that it became necessary to provide further accommodation. In 1910 the old Smallpox Hospital at Small Heath was converted into a sanatorium with eighty beds. Both these institutions are now being enlarged; when completed there will be accommodation for sixty-eight patients at Salterley Grange, and 220 at Small Heath. In addition to the above, provision has been made for the more advanced cases at West Heath Fever Hospital, where fifty-two beds will shortly be available. In 1912 the Committee of the Hospital Saturday Fund erected a sanatorium of 100 beds at Romsley Hill as a memorial to the late Sir William Cooke. The Public Health Committee lease ninety of these beds. When the extensions now in progress are completed Birmingham will have a total of 409 beds available for consumptive patients.

The question of cost is naturally a serious one. Public health authorities will now, however, receive considerable aid from the State to enable adequate provision to be made. Where municipal authorities decide to erect sanatoria, the State will contribute three-fifths of the total cost per bed, provided that the total outlay per bed does not exceed £150. Should, however, this amount be exceeded, the State will still contribute the maximum amount of £90 per bed. The cost of maintenance will be heavy, but here, too, the State steps in and helps by means of what is known as the Hobhouse grant. This consists in the payment of half the expenditure after deducting from the total cost of maintenance the amount received from the Local Insurance Committee for the treatment of insured people. This leaves only a comparatively small proportion of the total cost to be borne by the ratepayers. In the case of children's beds the Hobhouse grant is contingent upon certain elementary education being given to the patients.

The question of a suitable site for the erection of a sanatorium does not present great difficulties, as it is now recognised that it is not essential to build at a distance from populous centres. Excellent results have been obtained in institutions situated quite close to large cities. It is of importance, however, that the site selected should, where possible, be some distance above sea level, and that it should not be shut in by surrounding streets or buildings. Where such buildings do not already exist, it is advisable to take precautions against future building operations so enclosing the sanatorium that it becomes unsuitable for the purpose. Among other advantages claimed are diminished cost owing to greater accessibility; the possibility of utilising the town water supply, etc.

The success of each and every sanatorium depends, in the main, upon the character and ability of its medical staff. Dettweiler, the famous German physician, said that "the medical director of a sanatorium for consumptives should not take upon himself the responsibility of such a position unless he is fully prepared, and honestly feels, that he can excel his co-workers in strength, creative power, discretion, faithfulness, and duty." The medical head of a sanatorium must be a disciplinarian, but it must be "the iron hand in the velvet glove" type of discipline. As Dixon and Wynn have truly said, "the blame for the appointment of unsuitable men to superintend sanatoria must be largely accepted by the various committees of management; the salary offered has usually been totally inadequate, and the candidate appointed has been more often than not a recently qualified man without any experience either of pulmonary tuberculosis, sanatorium methods, or general practice, all of which are very necessary for the man who is to direct a sanatorium successfully."

Too frequently the remuneration and accommodation provided for the medical staff is inadequate. The medical superintendent of every sanatorium should be provided with a house and garden, situated within or in close proximity to the grounds. Public health authorities make a mistake in only catering for the unmarried resident. Where men are devoting their lives to a particular branch of medicine, it is too much to expect that they are to remain bachelors for all time. This principle has been recognised in the mental hospital service, though not to the extent desirable. Medical officers of health can do a great deal by urging these points upon their committees. Economy should not be obtained at the expense of the medical staff.

Efficient nursing is of great importance in sanatoria, and one must dispose of the idea—all too prevalent—that any nursing will do. Good nursing is of as much importance in a sanatorium as in a general hospital. Nurses should be of some social standing, as, where their social position is only just above that of the domestic servant, it will be found that the discipline is lax, and that there is a tendency for them to fraternise too readily with the patients. The number of nurses required at a sanatorium depends entirely upon the character of the buildings. Where these are on the cubicle system or consist largely of separate chålets, a larger number of nurses will be required than where the buildings are long pavilions open to inspection from either end. The City Sanatorium at Birmingham is on the latter plan, and it is found that one nurse to every eight beds is sufficient.

The method of working and the results to be aimed at will vary somewhat according to the number of beds and the type of patient to whom they are allotted. Where there is more than one sanatorium, it is possible to utilise each for a different class of patient; advanced cases may be treated quite apart from early ones. A larger sanatorium will receive all types of cases, except the very advanced, and either deal with the cases entirely or distribute them to other sanatoria. There will naturally be a certain amount of interchange between all the institutions. Some advanced cases, for example, may so improve under treatment at a home of rest that it will be possible to draft them to one of the other institutions.

### The Birmingham System.

The system adopted in Birmingham has given excellent results. All cases are dealt with in the first place at the tuberculosis centre—this being an integral part of the system. A certain proportion of patients can be dealt with throughout as out-patients; these come to the centre twice a week for tuberculin or other treatment. Those patients whose condition precludes their being treated as out-patients are sent to the City Sanatorium or that at Romsley Hill, where their average stay is six to eight weeks. They are usually kept in bed for the first week, and as soon as the temperature becomes normal, or nearly so, tuberculin is usually given. As soon as their condition warrants it, the patients are started on exercise and very light work. At the end of six to eight weeks the condition of some patients has so far improved that they can be discharged, and continue treatment as out-patients, and others, according to their progress and physical condition, are drafted to Salterley Grange Sanatorium for a further period of two or three months, or are recommended for domiciliary treatment if insured persons. Advanced cases are dealt with at West Heath Sanatorium. On their return from the various sanatoria all patients report themselves at the tuberculosis centre, and where necessary and advisable continue treatment as out-patients. A number of health visitors are employed, and visit the homes, and so keep in touch with the patients.

A great feature of the work at the sanatoria is the education of the patients in the methods adopted for the prevention of the disease. Individual instruction and lectures—some of which are open to patients' friends—are given in the essentials of treatment and prevention. It is difficult to over-estimate the value of this side of sanatorium work.

Municipal sanatoria will almost entirely be occupied by members of the working classes, and such patients, being largely unaccustomed to discipline, are apt to find the strict routine very irksome. An aversion to labour has been noted among individuals who have spent a few months in a sanatorium. In suitable cases light work in the open air, in addition to preparing the patient for the resumption of his ordinary occupation, has an excellent effect in keeping him interested and less liable to dwell upon the restrictions imposed upon him.

In many of the smaller towns and counties it will probably be found impossible to provide more than one institution, and for the sake of economy the authorities of adjoining districts may find it advisable to maintain a joint sanatorium. Modest substantial structures should be erected in order that the greatest number of patients may be received at a minimum of expense. Where accommodation for advanced cases has also to be provided, it is essential that the hospital block and its surrounding walks should be quite distinct from the other portions of the sanatorium, though it may be within the same ring fence and under the same medical superintendent. In estimating the number of beds required for a particular district it must not be overlooked that some provision must be made for "contacts." Where the yearly average of notifications is 500 for example, the number of contacts will be about 2,000. Some provision must be made for their early detection and treatment.

The question of diet in municipal sanatoria does not call for much comment. Plain, well cooked food is the main essential. An effort should also be made to instruct female patients particularly in the class of food to buy in their own homes. Certain articles of diet as porridge, beans, macaroni, rice, etc., are not appreciated at their proper value. If these are served to the patients during their stay in the sanatorium they are more likely to continue their use afterwards. Many patients expect to be served with large quantities of eggs during their stay. Besides being expensive, they are not necessary for those patients who are not confined to bed, whose appetites are good, and who can partake of the usual fare provided. Naturally, where the appetite is poor and digestion impaired extras will be ordered. It is usual at most sanatoria to grow a large proportion of the vegetables used. This makes for economy, and at the same time provides light work for patients in the nature of hoeing, weeding, etc.

Some mention should perhaps be made of amusements. Where the average stay of patients is only six to eight weeks, the provision of special amusements is not very necessary. It is certainly not justifiable to go to the expense of building special recreation halls as proposed by some authorities. Bowls form an easy and enjoyable game for those sufficiently well to take part. Draughts, dominoes, and similar games may be provided for use on the open verandahs. Card playing should be strictly prohibited, as it invariably leads to gambling among the type of patients in municipal sanatoria. Smoking is a vexed question. On the whole in institutions where the stay is comparatively short it is better to prohibit the use of tobacco. Its use tends to increase expectoration, and to subvert discipline.

